# Differential diagnosis of infectious diseases, drug-induced lung injury, and pulmonary infiltration due to underlying malignancy in patients with hematological malignancy using HRCT

**DOI:** 10.1007/s11604-022-01328-4

**Published:** 2022-09-09

**Authors:** Nobuyuki Tanaka, Yoshie Kunihiro, Reo Kawano, Toshiaki Yujiri, Kazuhiro Ueda, Toshikazu Gondo, Taiga Kobayashi, Tsuneo Matsumoto

**Affiliations:** 1 Department of Radiology, Yamaguchi University Graduate School of Medicine, 1-1-1 Minamikogushi, Ube, Yamaguchi 755-8505 Japan; 2 Center for Clinical Research, Yamaguchi University Graduate School of Medicine, 1-1-1 Minamikogushi, Ube, Yamaguchi 755-8505 Japan; 3 Department of Clinical Laboratory Sciences, Yamaguchi University Graduate School of Medicine, 1-1-1 Minamikogushi, Ube, Yamaguchi 755-8505 Japan; 4 Department of Surgery and Clinical Science, Yamaguchi University Graduate School of Medicine, 1-1-1 Minamikogushi, Ube, Yamaguchi 755-8505 Japan; 5 Division of Surgical Pathology, Yamaguchi University Hospital, 1-1-1 Minami-Kogushi, Ube, Yamaguchi 755-8505 Japan; 6 Yamaguchi Health and Service Association, 3-1-1 Yosiki-simohigashi, Ube, Yamaguchi 753-0814 Japan; 7 Present Address: Department of Radiology, National Hospital Organization, Yamaguchi-Ube Medical Center, 685 Higashikiwa, Ube, Yamaguchi 755-0241 Japan; 8 Present Address: Center for Integrated Medical Research, Hiroshima University Hospital, Kasumi 1-2-3 Minami-ku, Hiroshima, Hiroshima 734-8551 Japan; 9 Present Address: Department of General Thoracic Surgery, Kagoshima University Graduate School of Medicine, 8-35-1 Sakuragaoka, Kagoshima, 890-8520 Japan; 10 Present Address: Division of Surgical Pathology, UBE Kohsan Central Hospital, 750 Nishikiwa, Ube, Yamaguchi 755-0151 Japan

**Keywords:** Hematological malignancy, Infection, Drug-induced lung injury, Pulmonary infiltration due to underlying malignancy, High-resolution CT (HRCT)

## Abstract

**Purpose:**

To differentiate among infectious diseases, drug-induced lung injury (DILI) and pulmonary infiltration due to underlying malignancy (PIUM) based on high-resolution computed tomographic (HRCT) findings from patients with hematological malignancies who underwent chemotherapy or hematopoietic stem cell transplantation.

**Materials and methods:**

A total of 221 immunocompromised patients with hematological malignancies who had proven chest complications (141 patients with infectious diseases, 24 with DILI and 56 with PIUM) were included. Two chest radiologists evaluated the HRCT findings, including ground-glass opacity, consolidation, nodules, and thickening of bronchovascular bundles (BVBs) and interlobular septa (ILS). After comparing these CT findings among the three groups using the *χ*^2^test, multiple logistic regression analyses (infectious vs noninfectious diseases, DILI vs non-DILI, and PIUM vs non-PIUM) were performed to detect useful indicators for differentiation.

**Results:**

Significant differences were detected in many HRCT findings by the *χ*^2^ test. The results from the multiple logistic regression analyses identified several indicators: nodules without a perilymphatic distribution [*p* = 0.012, odds ratio (95% confidence interval): 4.464 (1.355–11.904)], nodules with a tree-in-bud pattern [*p* = 0.011, 8.364 (1.637–42.741)], and the absence of ILS thickening[*p* = 0.003, 3.621 (1.565–8.381)] for infectious diseases, the presence of ILS thickening [*p* = 0.001, 7.166 (2.343–21.915)] for DILI, and nodules with a perilymphatic distribution [*p* = 0.011, 4.256 (1.397–12.961)] and lymph node enlargement (*p* = 0.008, 3.420 (1.385–8.441)] for PIUM.

**Conclusion:**

ILS thickening, nodules with a perilymphatic distribution, tree-in-bud pattern, and lymph node enlargement could be useful indicators for differentiating among infectious diseases, DILI, and PIUM in patients with hematological malignancies.

## Introduction

During the past several decades, due to the progression of intensive chemotherapy and hematopoietic stem cell transplantation (HSCT), the therapeutic outcomes of patients with hematological malignancy have become more favorable. In addition to the fact that patients undergoing chemotherapy or HSCT are susceptible to infection and drug-induced lung injury (DILI), pulmonary infiltration due to underlying malignancy (PIUM) could be a possible disease in patients to whom HSCT or repeated chemotherapy is no longer effective [1–3]. These three types of complications are a major cause of morbidity and mortality and the differential diagnosis among these three entities seems to be a major concern for physicians because the therapeutic strategy for each entity is quite different; therefore, early diagnosis and treatment are important. However, distinguishing among these complications is often difficult due to nonspecific chest symptoms and radiologic findings. According to several reports, a chest radiograph could show normal findings in up to 10% of patients with confirmed lung diseases [2–4].

High-resolution computed tomography (HRCT) is superior to chest radiography for the diagnosis of acute lung diseases in immunocompromised patients [2, 4]; however, even HRCT often shows nonspecific findings, including airspace consolidation, ground-glass opacity (GGO), and nodules; therefore, accurate diagnoses with a high degree of confidence are limited except for some diseases [5].

In the current study, we aimed to differentiate among infectious diseases, DILI and PIUM using a relatively large number of patients with hematologic malignancies who underwent chemotherapy or HSCT.

## Materials and methods

### Patients

The review board of the institution, in which this work was carried out, approved this study. The requirement for the patient’s informed consent was waived due to the retrospective nature of this study.

**Fig. 1:**
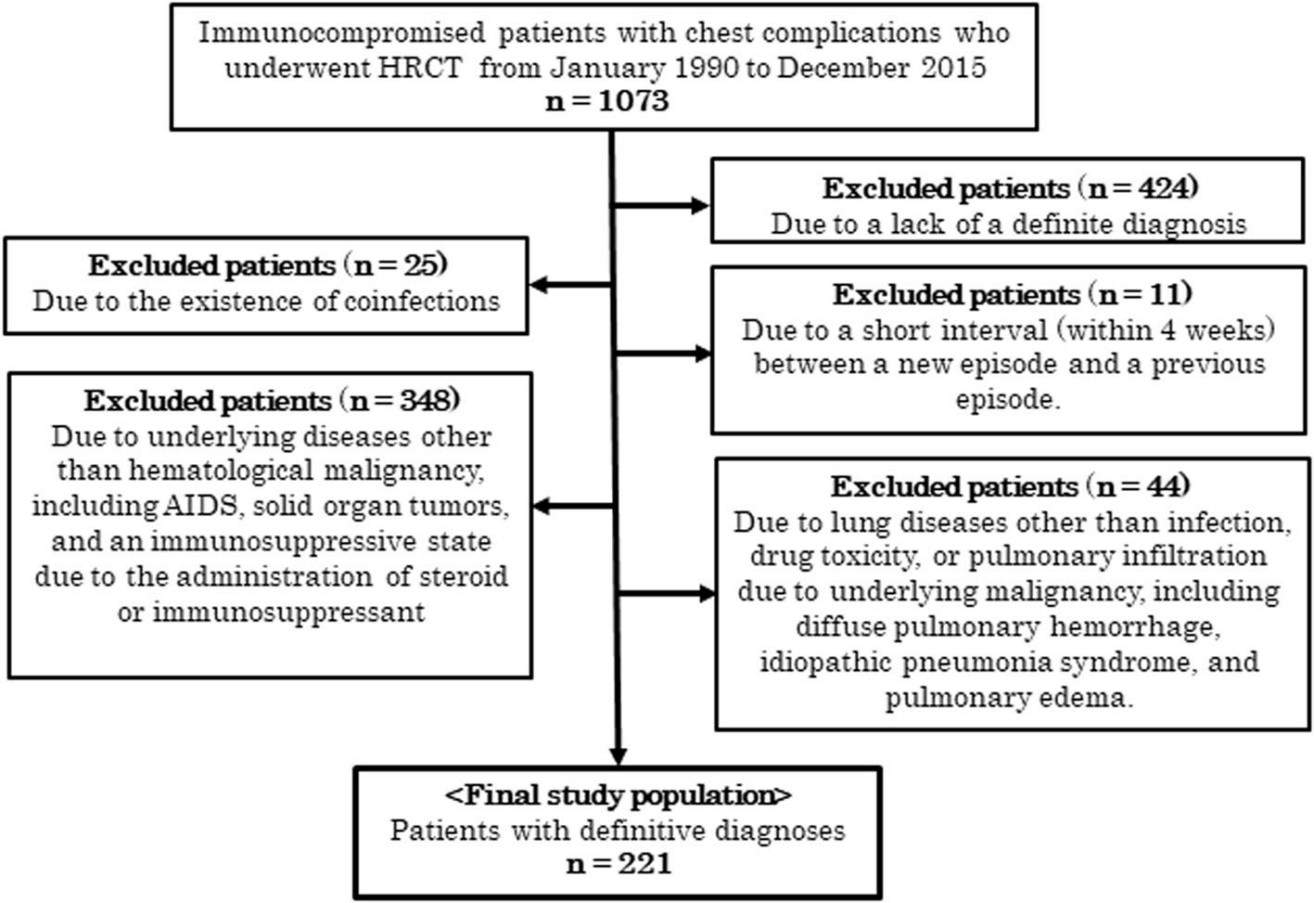
Flow diagram of patient selection

We retrospectively reviewed the CT database at this institution for acute chest complications in immunocompromised patients from January 1990 to December 2015. The selection criteria for this study were as follows: (1) HRCT was performed to investigate the lung abnormalities of the patients, (2) HRCT showed any parenchymal abnormalities, and (3) only one final diagnosis was determined for each case. First, we identified a total of 1073 immunocompromised patients with chest complications depicted on HRCT images. Among them, 852 patients were excluded due to lack of a definitive diagnosis (*n* = 424), coinfections (*n* = 25), a short interval (within four weeks) between a new episode and a previous episode (*n* = 11), underlying diseases other than hematological malignancy and not undergoing chemotherapy or HSCT (*n* = 348), and lung diseases other than infection, DILI, or PIUM (*n* = 44), including diffuse pulmonary hemorrhage, idiopathic pneumonia syndrome, and pulmonary edema; therefore, 221 cases were included in this study. The reason for exclusion due to a short interval was that the previous episode may have influenced the HRCT findings of the current episode. Finally, a total of 221 cases of 188 patients [129 males and 92 females; mean age was 49.6 standard deviation (SD),17.0; age range: 5–82 years] were identified as the current study population, as shown in Fig. 1. Among the 188 patients, 25 patients had 2 episodes, and 4 patients had 3 episodes of complications, resulting in a total of 221 cases. The final diagnoses of the 221 cases included 141 cases of infectious diseases, 24 cases of DILI and 56 cases of PIUM; detailed information, including the diagnostic methods, is shown in Table 1. Some cases of DILI and PIUM were diagnosed based on the clinical course. Criteria for diagnosis of DILI due to clinical course included onset of lung diseases after chemotherapy, immediate responsiveness to newly administered steroids, and exclusion of infections from the results of sputum culture, serum antigens or antibodies for specific microorganisms or bronchoalveolar lavage. Criteria for diagnosis of PIUM due to clinical course included responsiveness of lung diseases to the administration of chemotherapy. The diagnoses of these cases were basically determined by the attending physicians according to detailed analyses of all physical and laboratory findings and the responsiveness to the therapy including steroids/immunosuppressants and chemotherapy. The underlying diseases of 188 patients were leukemia (*n* = 99), malignant lymphoma (*n* = 62), and other hematological malignancies (*n* = 27) including myelodysplastic syndrome and multiple myeloma.

**Table 1:** Characteristics of all 221 cases and diagnostic methods for all cases included in this study

Lung disease groups	Age [mean (SD)]	Gender	Diagnostic methods
Infectious diseases (*n* = 141) Bacterial pneumonia 50 Fungal infection 34 Septic emboli 7 Tuberculosis 3 Pneumocystis pneumonia 40 Cytomegalovirus pneumonia 7	48.5 (17.2) (5–81 years)	Male 86 Female 55	Sputum smear/culture 32 Blood culture 28 Serum antigen/antibody 33 Urinary antigen 1 BAL 30 TBLB 7 BAL + TBLB 1 SLB 1 Autopsy 8
Drug toxicity (*n* = 24)	56.9 (15.0) (20–82 years)	Male 12 Female 12	DLST 2 TBLB 2 BAL + TBLB 2 CT guided biopsy 1 Clinical course and laboratory findings 17
Pulmonary infiltration due to underlying malignancy (*n* = 56) Leukemic infiltration 25 Lymphoma infiltration 31	49.3 (17.0) (20–78 years)	Male 31 Female 25	BAL 5 TBLB 3 BAL + TBLB 1 CT guided biopsy 1 SLB 3 Autopsy 4 Cytology of pleural effusion 5 Clinical course and laboratory findings 34

*SD* standard deviation, *BAL *bronchoalveolar lavage, *TBLB* transbronchial lung biopsy, *SLB *surgical lung biopsy, *DLST *drug lymphocyte stimulation test

### CT scanning

CT scans were obtained using a TCT-900S (Canon medical systems corporation, Tokyo, Japan), a Somatom Plus 4, a Volume Zoom, Somatom Definition and Somatom Sensation 64 (Siemens, Erlangen, Germany). CT scans were obtained at suspended end-inspiration in the supine position without intravenous contrast material. The CT protocols were diverse due to the long period of the current study. With the TCT-900S scanner, which is not a multislice CT scanner, after 10 mm collimation scans were obtained at contiguous 10-mm intervals through the entire chest, all patients underwent HRCT through the region showing abnormal parenchymal findings at 2 mm collimation. With other multislice CT scanners, after contiguous 10, 7 or 5 mm slice CT images were obtained through the chest, additional 1 or 2 mm slice HRCT images were obtained at 1, 2, 5, or 10 mm intervals through the abnormal lung parenchyma. The scanning parameters were 140 kVp and 160–250 effective mAs. All images were viewed at the lung (window width, 1500 or 1750 HU; window level, -600 or -700 HU) and mediastinal (window width, 250–400 HU; window level, 40–50 HU) windows.

### Interpretation of CT images

The CT images were independently evaluated by two chest radiologists (16 and 29 years of experience) in random order without knowledge of the clinical information of the patients except that they were all patients with hematological malignancies who underwent chemotherapy or HSCT. Cases of discordant results between the two radiologists were resolved by consensus of the same two radiologists.

Each of the following HRCT findings was separately assigned as “present” or “absent”: (a) consolidation, (b) GGO, (c) a crazy-paving pattern, (d) a mosaic pattern, (e) nodules, (f) nodules with a halo sign, (g) nodules with a tree-in-bud (TIB) pattern, (h) bronchovascular bundle (BVB) thickening, (i) interlobular septal (ILS) thickening, (j) hilar or mediastinal lymph node (LN) enlargement, and (k) pleural effusion. Concerning the three HRCT findings of (a), (b) and (e), the extent of the lesions within the entire bilateral lung field was graded subjectively on a five-point scale (0: 0%, 1: 1–25%, 2: 26–50%, 3: 51–75%, and 4: 76–100%). In addition, the overall lesional extent within the entire lung field was also graded on the same five-point scale.

When GGO or consolidation was present, the predominance of consolidation or GGO (GGO/Con predominance) was classified as GGO predominance, consolidation predominance, or their equal predominance of both. The distribution of GGO or consolidation (GGO/Con distribution) was classified as segmental, nonsegmental, or lobular. Nodules were classified by size as micro (< 3 mm), small (> 3 mm and < 10 mm), or large (> 10 mm), and by distribution as centrilobular, perilymphatic, or random.

Furthermore, the overall lesional distribution was classified axially (axial distribution) as inner, outer, diffuse or indeterminate and craniocaudally (craniocaudal distribution) as upper, lower, diffuse or indeterminate. One predominant CT pattern was recorded for each patient as follows: a micro or small (micro/small) nodular pattern, a large nodular pattern, a diffuse GGO pattern, a segmental GGO or consolidation (GGO/Con) pattern, a nonsegmental (nonseg) GGO/Con pattern, or a BVB or ILS thickening (BVB/ILS) pattern.

### Statistical analysis

Interobserver agreement between the two radiologists was evaluated by calculating the kappa value (κ-value) as follows: poor (*κ* = 0.00–0.20), fair (*κ* = 0.21–0.40), moderate (*κ* = 0.41–0.60), good (*κ* = 0.61–0.80), or excellent (*κ* = 0.81–1.00) [7]. The extent of consolidation, GGO, nodules, and overall distribution were calculated using weighted kappa statistics.

Each CT finding and CT pattern were compared among the three groups using a Chi-square (*χ*^2^) test for independence. A comparison of the extent of CT findings among the three groups was performed using the Kruskal–Wallis test. A *p* value less than 0.05 was considered indicative of a significant difference. Furthermore, as an index of the degree of significant differences, Pearson’s *χ*^2^ value was used. When the *χ*^2^ test detected a significant difference among the groups, adjusted standardized residuals were calculated to identify the groups for which the CT findings contributed to the significant difference. An adjusted standardized residual of > 1.96 or < −1.96 was considered indicative of a group with a significantly higher or lower frequency, respectively.

Multiple logistic regression analyses were conducted to identify significant indicators for the differentiation of each group from the other two groups, for example, between infectious diseases and the two other diseases, including DILI and PIUM. The forward selection (likelihood ratio) method was used for the multiple logistic regression analyses, and all HRCT findings, including parametric factors (extent of HRCT findings), were included. All statistical analyses were performed using commercially available software (SPSS, version 24.0, IBM).

## Results

The results of the comparison of HRCT findings among the three groups using the *χ*^2^ test and interobserver variabilities are shown in Table 2. Regarding the interobserver variabilities, the κ-values were generally moderate except for the κ-values for the GGO/Con distribution (fair: *κ* = 0.263, nodules with TIB (fair: *κ* = 0.265) and the overall axial distribution (fair: *κ* = 0.354). The *χ*^2^ test showed significant differences in more than half of the HRCT findings. The *χ*^2^ values were especially high for the GGO/Con distribution (25.93), the distribution of nodules (30.65) and the predominant CT pattern (36.14).

**Table 2:** Comparison of the HRCT findings among the three groups

		Infectious disease (*n* = 141)	DILI (*n* = 24)	PIUM (*n* = 56)	*P* value	*χ*^2^ value	*κ *value
Consolidation (%)	–	90 (64)	14 (58)	30 (54)	0.401	1.827	0.518
Extent (SD)	–	0.78 [0.65]	0.69 (0.64)	0.72 [0.77]	0.691^+^	–	0.570^$^
GGO (%)	–	128 (91)	23 (96)	44 (79)*	0.03	7.25	0.540
Extent (SD)	–	1.86 [1.16] ±	2.38 (1.09)	1.45 [1.08]	0.004^+^	–	0.673^$^
Crazy-paving (%)	–	49 (35)**	5 (21)	7 (13)*	0.005	10.55	0.365
Mosaic pattern (%)	–	44 (31)	10 (42)	7 (13)*	0.008	9.68	0.568
GGO/con predominance (%)	Con. pred	44 (34)	3 (13)	12 (27)	0.151	6.73	0.540
	GGO pred	78 (60)	20 (87)	30 (67)			
	Equal	8 (6)	0 (0)	3 (7)			
GGO/con distribution (%)	Segmental	57 (44)**	3 (13)*	14 (31)	< 0.001	25.93	0.263
	Nonseg	23 (18)*	8 (35)	23 (51)**			
	Lobular	50 (38)	12 (52)	8 (18)*			
Nodule (%)	–	86 (61)	10 (42)	39 (70)	0.063	5.53	0.469
Extent [SD]	–	0.89 [0.92]	0.92 [1.32]	1.15 [1.05]	0.207^+^	–	0.568^$^
Size (%)	Micro	21 (24)	6 (60)**	10 (26)	0.021	11.56	0.468
	Small	31 (36)	3 (30)	7 (18)*			
	Large	34 (40)	1 (10)*	22 (56)**			
Distribution (%)	Centrilobular	40 (47)**	7 (70)**	6 (15)*	< 0.001	30.65	0.571
	Perilymphatic	7 (8)*	0 (0)	17 (44)**			
	Random	39 (45)	3 (30)	16 (41)			
Nodule with halo (%)	–	36 (26)	1 (4)	12 (21)	0.066	5.45	0.543
Nodule with TIB (%)	–	22 (16)**	1 (4)	1 (2)*	0.010	9.15	0.265
BVB thickening (%)	–	60 (43) *	10 (42)	36 (54)**	0.018	8.01	0.621
ILS thickening (%)	–	48 (34)*	20 (83)**	30 (54)	< 0.001	22.78	0.579
Axial distribution (%)	Inner	7 (5)	0 (0)	8 (14)**	0.006	18.25	0.354
	Outer	38 (27)	5 (21)	12 (21)			
	Diffuse	46 (33)	16 (67)**	17 (30)			
	Indeterminate	50 (35)	3 (13)*	19 (34)			
Craniocaudal distribution (%)	Upper	16 (11)	1 (4)	10 (18)	0.076	11.42	0.408
	Lower	31 (22)	8 (33)	10 (18)			
	Diffuse	48 (34)	13 (54)	18 (32)			
	Indeterminate	46 (33)	2 (8)	18 (32)			
Overall distribution [SD] (SD)	–	2.40 [1.00]	2.81 [0.88]	2.34 [1.01]	0.125^+^	–	0.571^$^
Predominant CT pattern (%)	Micro/small nodule	21 (15)	2 (8)	10 (18)	< 0.001	36.14	0.510
	Large nodule	24 (17)	1 (4)*	18 (32)**			
	Diffuse GGO	39 (28)	11 (46)**	8 (14)*			
	Segmental GGO/Con	44 (31)**	3 (13)	6 (11)*			
	Nonseg. GGO/Con	12 (9)*	5 (21)	10 (18)			
	BVB/ILS thickening	1 (1)*	2 (8)	4 (7)**			
LN enlargement (%)	–	30 (21)*	5 (21)	30 (54)**	< 0.001	21.09	0.709
Pleural effusion (%)	–	38 (27)	3 (13)	18 (32)	0.190	3.33	0.733

All values are presented as the number of cases, with percentages in parentheses

*DILI *drug-induced lung injury, *PIUM *pulmonary infiltration due to underlying malignancy, *Cons *consolidation, *GGO* ground-glass opacity, *TIB* tree-in-bud, *LN *lymph node, *Hematol. malig* hematological malignancy, *pred* predominance, *Nonseg* nonsegmental, *BVB* bronchovascular bundle, *ILS* interlobular septum,* SD* standard deviation

^+^Kruskal–Wallis test

^$^Weighted κ(kappa) value

* and **Chi-square (*χ*^2^) test for independence

*Significantly less frequent (adjusted standard residual < −1.96)

**Significantly more frequent (adjusted standard residual > 1.96)

Table 3 shows the detailed frequencies of HRCT findings among infectious diseases. The HRCT findings did not share the same high or low frequency among individual diseases; for example, the frequency of ILS thickening in infectious diseases was significantly higher in pneumocystis pneumonia (PCP) (45%) and cytomegalovirus (CMV) pneumonia (57%) than in other infectious diseases.

**Table 3:** Detailed frequencies of HRCT findings among infectious diseases

		Infectious disease (*n* = 141)					
		Bacterial-P (*n* = 50)	Fungal-I (*n* = 34)	PCP (*n* = 40)	CMV (*n* = 7)	Septic emboli (*n* = 7)	TB (*n* = 3)
Consolidation (%)	–	40 (80)	18 (53)	19 (48)	5 (71)	5 (71)	3 (100)
GGO (%)	–	46 (92)	25 (74)	40 (100)	7 (100)	7 (100)	3 (100)
Crazy-paving (%)	–	18 (36)	8 (24)	19 (48)	4 (57)	0 (0)	0 (0)
Mosaic pattern (%)	–	8 (16)	3 (9)	29 (73)	3 (43)	0 (0)	1 (33)
GGO/con predominance (%)	Con. pred	25 (53)	11 (42)	1 (3)	1 (14)	4 (57)	2 (67)
	GGO pred	18 (38)	12 (46)	39 (98)	5 (71)	3 (43)	1 (33)
	Equal	4 (9)	3 (12)	0 (0)	1 (14)	0 (0)	0 (0)
GGO/con distribution (%)	Segmental	33 (70)	17 (65)	1 (3)	2 (29)	2 (29)	2 (67)
	Nonseg	6 (13)	7 (27)	3 (8)	2 (29)	5 (71)	0 (0)
	Lobular	8 (17)	2 (8)	36 (90)	3 (43)	0 (0)	1 (33)
Nodule (%)	–	30 (60)	30 (88)	11 (28)	5 (71)	7 (100)	3 (100)
Size (%)	Micro	10 (33)	5 (17)	3 (27)	1 (14)	1 (14)	1 (33)
	Small	9 (30)	9 (30)	5 (46)	3 (43)	3 (43)	2 (67)
	Large	11 (37)	16 (53)	3 (27)	1 (14)	3 (43)	0 (0)
Distribution (%)	Centrilobular	17 (57)	11 (37)	7 (64)	3 (43)	0 (0)	2 (67)
	Perilymphatic	2 (7)	2 (7)	0 (0)	1 (14)	2 (29)	0 (0)
	Random	11 (36)	17 (57)	4 (36)	1 (14)	5 (71)	1 (33)
Nodule with halo (%)	–	12 (24)	15 (44)	2 (5)	4 (57)	3 (43)	0 (0)
Nodule with TIB (%)	–	10 (20)	7 (21)	2 (5)	2 (29)	0 (0)	1 (33)
BVB thickening (%)	–	30 (60)	16 (47)	6 (15)	2 (29)	4 (57)	2 (67)
ILS thickening (%)	–	19 (36)	3 (9)	18 (45)	4 (57)	3 (43)	1 (33)

All values are presented as the number of cases, with percentages in parentheses

*Bacterial-P *bacterial pneumonia, *Fungal-I *fungal infection, *TB *tuberculosis, *PCP* pneumocystis pneumonia, *CMV-P *cytomegalovirus pneumonia,* GGO* ground-glass opacity, *Cons *consolidation, *TIB* tree-in-bud, *BVB* bronchovascular bundle, *ILS* interlobular septum, *LN* lymph node, *pred* predominance, *Nonseg* nonsegmental

**Fig. 2:**
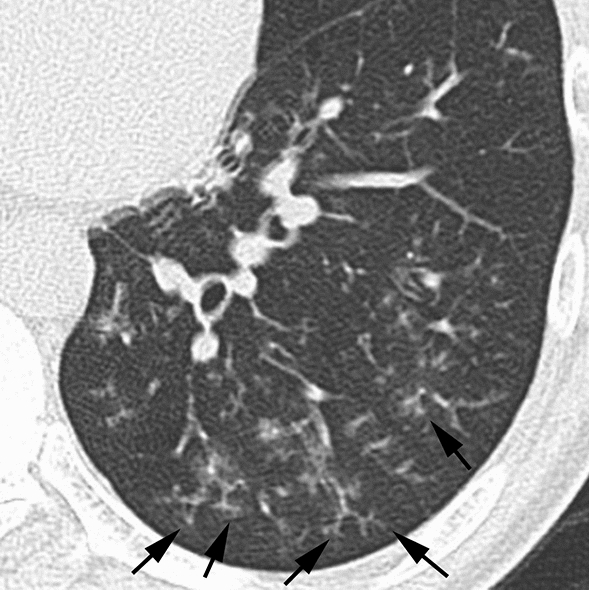
Infectious disease (bacterial pneumonia) in a 56-year-old male patient with acute myelogenous leukemia**. **HRCT image of the left lower lung showing nodules with a centrilobular distribution and TIB pattern (arrows). Note that ILS thickening is not evident

**Fig. 3:**
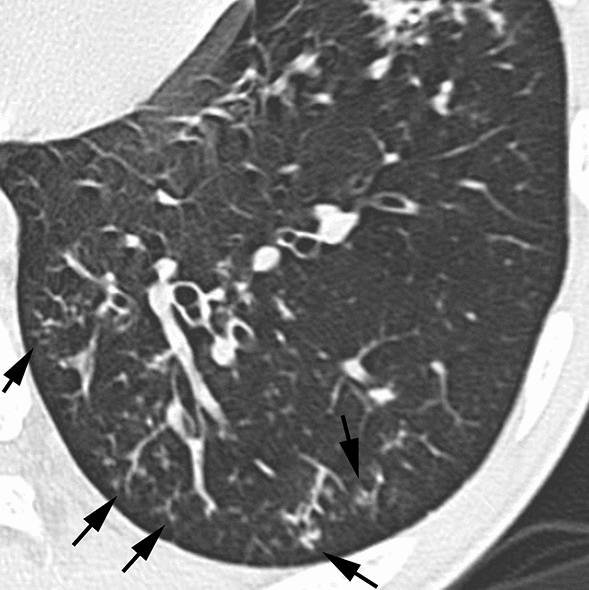
Infectious disease (invasive aspergillosis) in an 18-year-old male patient with acute myelogenous leukemia**. **HRCT image of the left lower lung showing nodules with a centrilobular distribution and TIB pattern (arrows). Note that ILS thickening is not evident

**Fig. 4:**
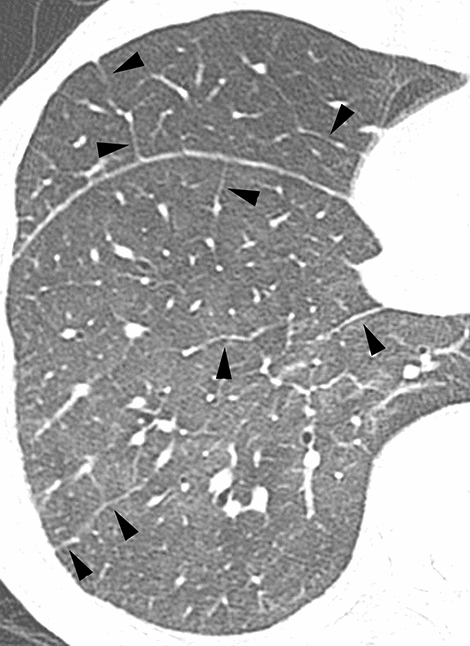
Drug-induced lung injury in a 65-year-old male patient with multiple myeloma.HRCT image of the right lower lung showing extensive GGO and ILS thickening (arrowheads). Note also that nodules are not evident

**Fig. 5:**
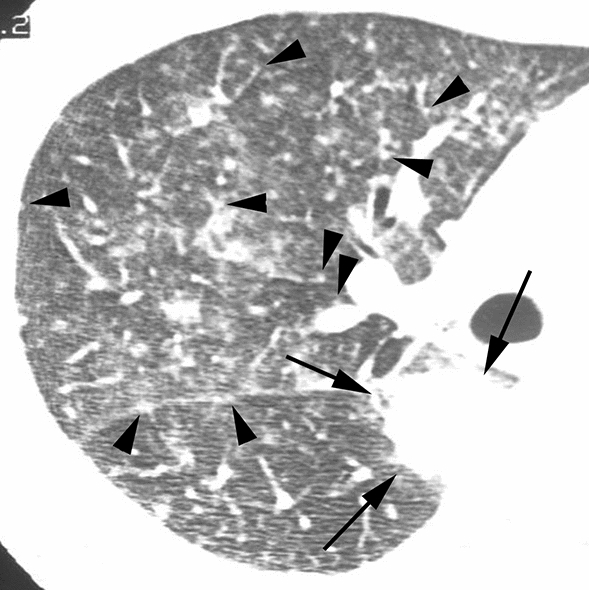
Pulmonary infiltration due to underlying malignancy (leukemic infiltration) in a 44-year-old male patient with acute myelogenous leukemia.HRCT image of the right middle lung showing multiple nodules varying in size. Note that small nodules are attached to the pleura or thickened BVB (arrowheads), with differences in the number of nodules in the segments, reflecting a perilymphatic distribution rather than a random distribution. Note also that a large nodule is observed in the right lower lobe (arrows)

**Fig. 6:**
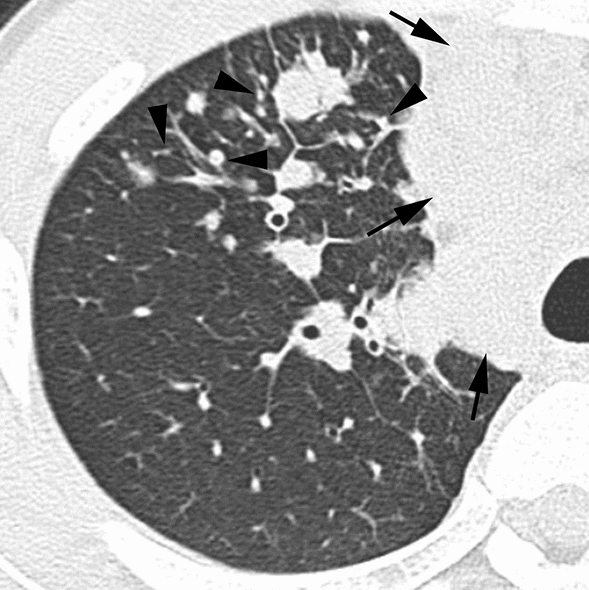
Pulmonary infiltration due to underlying malignancy (lymphoma infiltration) in a 23-year-old male patient with non-Hodgkin lymphoma. HRCT image of the right upper lung showing multiple nodules varying in size (arrowheads), some of which attached to the pulmonary arteries, veins, or ILS, reflecting a perilymphatic distribution. Note the enlargement of the mediastinum with LN enlargement (arrows), which is not clear due to the lung window setting of this CT image

According to the results of the multiple logistic regression analyses, several significant indicators were detected for each group (Table 4). In infectious diseases, nodules without a perilymphatic distribution, nodules with a TIB pattern, and the absence of ILS thickening (Figs. 2, 3) were detected as useful indicators for differentiation. Among these indicators, the significance of nodules with a TIB pattern might be degraded due to low sensitivity (15.6%). Instead, the specificity (97.5%) and positive predictive value (PPV) (91.7%) for this indicator were the highest among these indicators detected by multiple logistic regression analyses. In DILI, the presence of ILS thickening (Fig. 4) was detected as a useful indicator for differentiation. In PIUM, nodules with a perilymphatic distribution and the presence of LN enlargement (Figs. 5, 6) were detected as useful indicators for differentiation.

**Table 4:** Three multiple logistic regression analyses for differentiating one disease from the others.

A. Differentiation of infectious diseases from other diseases										
	Infectious diseases (*n* = 141)	Others (*n* = 80)	Wald value	Odds ratio [95% CI]	*P* value	Sensitivity	Specificity	Accuracy	PPV	NPV
Nodular distribution										
Perilymphatic Others	7 (8) 79 (92)	17(35) 32(65)	6.287	– 4.464 [1.355, 11.904]	0.012	91.9%	34.7%	71.1%	71.2%	70.8%
Tree-in-bud										
Positive Negative	22 (16) 119 (84)	2 (3) 78 (98)	6.512	8.364 [1.637, 42.741] –	0.011	15.6%	97.5%	45.2%	91.7%	39.6%
ILS thickening										
Positive Negative	48 (34) 93 (66)	50 (63) 30 (38)	9.032	– 3.621 [1.565, 8.381]	0.003	66.0%	62.5%	64.7%	75.6%	51.0%

B. Differentiation of DILI from other diseases										
	DILI (*n* = 24)	Others (*n* = 197)	Wald value	Odds ratio [95% CI]	*P* value	Sensitivity	Specificity	Accuracy	PPV	NPV
ILS thickening										
Positive Negative	20 (83) 4 (17)	78 (40) 119 (60)	11.921	7.166 [2.343, 21.915] –	0.001	83.3%	60.4%	62.9%	20.4%	96.7%

C. Differentiation of PIUM from other diseases										
	PIUM (*n* = 56)	Others (*n* = 165)	Wald value	Odds ratio [95% CI]	*P* value	Sensitivity	Specificity	Accuracy	PPV	NPV
Nodular distribution										
Perilymphatic Others	17 (44) 22 (56)	7 (7) 89 (93)	6.497	4.256 [1.397,12.961] –	0.011	43.6%	92.7%	78.5%	70.8%	80.2%
LN enlargement										
Positive Negative	30 (54) 26 (46)	35 (21) 130 (79)	7.114	3.420 [1.385, 8.441] –	0.008	53.6%	78.8%	72.4%	46.2%	83.3%

All values in parentheses show percentages.

*DILI *drug-induced lung injury, *PIUM *pulmonary infiltration due to underlying malignancy, *ILS* interlobular septum, *BVB* bronchovascular bundle, *LN* lymph node

*PPV* positive predictive value, *NPV* negative predictive value

Considering the highest *χ*^2^ values of a predominant CT pattern (36.14), the predominant CT pattern might be worth adding to the useful CT findings even though it was not detected as a significant indicator according to the multiple logistic regression analyses; a segmental GGO/Con pattern is frequent in infectious diseases, a diffuse GGO pattern is common in DILI, and large nodules or a BVB/ILS thickening pattern is frequently found in PIUM (Table 2). Furthermore, concerning the extent of HRCT findings, the extent of GGO was only detected as a significant factor by the Kruskal–Wallis test; DILI showed the highest extent of GGO, which might also be worth adding to the useful CT findings (Fig. 4).

## Discussion

It should be noted that all 221 subjects included in the current study were previously used in another study of 555 subjects that compared HRCT findings between infectious and noninfectious diseases [6]; however, the current study included only patients with hematological malignancies after chemotherapy or HSCT, while the previous report included immunocompromised patients other than hematological malignancies. Furthermore, while the previous report compared HRCT findings between patients with infectious diseases and noninfectious diseases, the current study compared those among infectious diseases, DILI, and PIUM; therefore, the purpose of the current study differs from that of the previous report.

According to the results of the multiple logistic regression analyses, ILS thickening is a significant indicator for the differentiation of infectious disease and DILI; the absence of ILS thickening is an indicator that differentiates infectious disease from the other two entities, while the presence of ILS thickening is also an indicator that differentiates DILI from the other two entities. Nodules with a perilymphatic distribution are also a significant indicator for the differentiation of infectious diseases and PIUM; nodules without a perilymphatic distribution are an indicator differentiating infectious diseases from the other two entities, while nodules with a perilymphatic distribution are also an indicator differentiating PIUM from the other two entities.

ILS thickening is observed in several interstitial lung diseases [8, 9], and has also been reported as a frequent HRCT finding in DILI [8, 10, 11]. According to two reports, ILS thickening was observed in 67% and 75% of patients with DILI [10, 11]. ILS thickening was also more frequent in PIUM, although the difference was not statistically significant. ILS thickening has been reported as a relatively frequent finding in leukemic infiltration; its frequency has been reported to be 33% [12], 55% [13], and 100% [14]. On the other hand, ILS thickening has not been a frequent HRCT finding for infectious diseases except for PCP and CMV pneumonia [15, 16]. In bacterial pneumonia or fungal infection, ILS thickening has been reported as an infrequent finding; according to the two reports, the frequency of ILS thickening in bacterial pneumonia and fungal infection was 0% for both diseases in one report [5] and 5 and 0%, respectively, in another report[17]. Concerning PCP and CMV pneumonia, ILS thickening has been reported as a relatively frequent finding (40–50%) in PCP and CMV pneumonia in two reports [15, 16]. In fact, ILS thickening was relatively frequent in PCP and CMV pneumonia in the current study (45 and 57%, respectively). Therefore, PCP or CMV pneumonia as well as noninfectious diseases should be considered as a possible diagnosis when ILS thickening is observed. Concerning the mechanisms of ILS thickening in PCP or CMV pneumonia, it is speculated that this finding may reflect the thickening of the interstitium or dilated lymphatics resulting from the organization of intraalveolar exudates [18]. However, since ILS thickening has not been reported as a predominant HRCT finding for PCP or CMV pneumonia [19–27] and other characteristic HRCT findings exist in these two entities [15, 16, 22, 26, 27], we believe that it is not so difficult to differentiate these two entities from DILI.

Nodules with a perilymphatic distribution correspond to infiltration of malignant cells or inflammatory cells or the presence of granulomatous lesions along or adjacent to the lymphatic channel within the lung, which sometimes creates nodular lesions and could be evident in lymphangitic carcinomatosis, leukemic or lymphoma infiltration, lymphocytic interstitial pneumonia, amyloidosis, and sarcoidosis. In leukemic or lymphoma infiltration, this finding and BVB thickening are frequently observed [13, 14, 28]. In infectious diseases, nodules without a perilymphatic distribution, namely, nodules with a centrilobular or random distribution, are frequently observed, with a centrilobular distribution often observed in bacterial pneumonia, fungal infection, CMV pneumonia, and tuberculosis, and with a random distribution often observed in CMV pneumonia and miliary tuberculosis [18].

In infectious diseases, in addition to the indicators of nodules without a perilymphatic distribution and the absence of ILS thickening, nodules with a TIB pattern were detected as a significant indicator according to the multiple logistic regression analyses. In the report by Okada et al., who dealt with 533 patients with centrilobular findings, the presence of centrilobular nodules with a TIB pattern strongly suggested the likelihood of infectious diseases [29]. However, the significance of nodules with a TIB pattern might be deducted due to low sensitivity (15.6%). One of the reasons for this low sensitivity is that the infectious disease group is not a uniform group. In fact, nodules with a TIB pattern are not frequent findings in PCP and septic emboli. Despite the low sensitivity of this finding, the specificity (97.5%) and PPV (91.7%) were the highest among the indicators detected by the multiple logistic regression analyses. Therefore, once this finding is detected, the possibility of infectious diseases may increase.

In DILI, in addition to ILS thickening detected as an indicator for its differentiation from the other two entities by multiple logistic regression analyses, GGO was more extensive in DILI than in the other two entities according to the Kruskal–Wallis test. In DILI, several HRCT patterns, including diffuse alveolar damage (DAD), nonspecific interstitial pneumonia (NSIP), hypersensitivity pneumonia (HP), organizing pneumonia and eosinophilic pneumonia patterns, have been reported [11, 30–33]. In these HRCT patterns, particularly in the DAD, HP, and NSIP patterns, extensive GGO is a characteristic HRCT finding, which might support the results of more extensive GGO in patients with DILI.

In PIUM, including lymphoma infiltration and leukemic infiltration, multiple logistic regression analysis detected nodules with a perilymphatic distribution as a significant indicator. In these diseases, pathological analyses have revealed that malignant cells infiltrate along or adjacent to the lymphatic channels along the bronchovascular bundles and pulmonary veins within the lungs, corresponding to the HRCT findings of nodules with a perilymphatic distribution. LN enlargement is also a key indicator discriminating PIUM from the other two entities according to multiple logistic regression analyses. The frequencies of LN enlargement in lymphoma infiltration and leukemic infiltration were relatively high (35% [34] and 55% [13], respectively). Thus, PIUM exhibited two HRCT findings that were associated with the lymphatic route within the lung.

Furthermore, several predominant CT patterns were detected according to the *χ*^2^ test results, although these were not detected as significant indicators according to the multiple logistic regression analyses: the segmental GGO/Con pattern was frequent in infectious diseases, diffuse GGO was often found in DILI, and large nodules and a BVB/ILS thickening pattern were common in PIUM. These patterns seem to reflect characteristic HRCT findings detected according to the multiple logistic regression analyses and *χ*^2^ test results, as mentioned above.

The current study has several limitations. First, the nature of this study was retrospective; therefore, the CT protocols and diagnostic procedures were diverse. Second, we divided the chest complications of these patients into three categories even though infectious diseases were diverse. Each disease in the infectious disease group has characteristic findings; therefore, the infectious disease group is a heterogeneous group. Establishing an inclusive group of infectious diseases consisting of several different entities may be controversial. However, it might be valuable to detect three indicators by multiple logistic regression analysis despite the heterogeneity of an infectious group. Third, the number of patients with DILI was relatively small compared with the numbers of patients with the other two entities. The diagnosis of DILI remains difficult and depends on the exclusion of other causes. No true gold standard test is available, and no specific histologic appearance is useful for the confirmation of the diagnosis, which seems to be the main reason why we had only a small number of patients with DILI. Fourth, the sensitivity of some indicators that were detected using multiple logistic regression analysis, including nodules with tree-in-bud patterns in infectious diseases, nodules with a perilymphatic distribution and LN enlargement in PIUM, was relatively low. This is a disadvantageous problem in this study. Instead, the specificity of these indicators was relatively high; therefore, once these findings are detected, the possibility of these diseases may increase. Finally, several other important diseases that should be considered as a possible diagnosis in clinical practice, including pulmonary hemorrhage, pulmonary edema, and idiopathic pneumonia syndrome, were excluded from this study because, this time, we aimed to differentiate only among three groups of infectious diseases, DILI, and PIUM. The results might have changed if these diseases had been included in this study.

Diagnosing chest complications in patients with hematological malignancies is not often straightforward, and one of the main concerns for clinicians who treat chest complications in such patients might be whether the pathology is an infectious disease, DILI, or PIUM. We detected several HRCT indicators among the three entities using a relatively large number of patients. To the best of our knowledge, this study dealt with the largest number of patients in the differential diagnosis among infectious diseases, DILI, and PIUM, which might be the essential advantageous point in the current study.

In conclusion, according to the statistical analyses including multiple logistic regression analyses, several HRCT findings were detected as characteristic for differentiating among infectious diseases, DILI, and PIUM in 221 patients with hematological malignancies who underwent chemotherapy or HSCT: nodules without a perilymphatic distribution, nodules with a TIB pattern, and the absence of ILS thickening in infectious diseases; the presence of ILS thickening and a wider extent of GGO in DILI; and nodules with a perilymphatic distribution and the presence of LN enlargement in PIUM.

## Declarations

We declare that this work has not been published previously, that it is not under consideration for publication elsewhere, that its publication is approved by all authors and tacitly or explicitly by the responsible authorities where the work was carried out, and that, if accepted, it will not be published elsewhere in the same form, in English or in any other language, including electronically without the written consent of the copyright-holder
